# Calculation procedure for RITY—A phenology model of *Ips typographus*

**DOI:** 10.1016/j.mex.2020.100845

**Published:** 2020-02-29

**Authors:** Nikica Ogris

**Affiliations:** Slovenian Forestry Institute, Večna pot 2, 1000 Ljubljana, Slovenia

**Keywords:** European spruce bark beetle, Phenology, Ecological modelling, Voltinism, Population dynamics

## Abstract

The RITY-2 phenology model was developed for the spatiotemporal simulation of the seasonal development of European spruce bark beetle, *Ips typographus*. RITY-2 is based on the PHENIPS model and was developed through improving PHENIPS with innovative approaches and calibrating and validating it for Slovenia. RITY-2 predictions are based on air temperatures from Integrated Nowcasting through a Comprehensive Analysis (INCA) system, which is used to calculate the effective bark temperature for beetle development. In this paper we describe the calculation procedure for RITY-2.

• INCA enables high resolution spatial and temporal simulations and predictions.
• An innovative procedure was introduced that finds the most appropriate spring date threshold from which the calculation of the phenological model is initiated.
• Simplified and customized linear models for calculation of the air temperature in the forest and bark temperatures were developed.

## Graphical abstract

**Figure:**
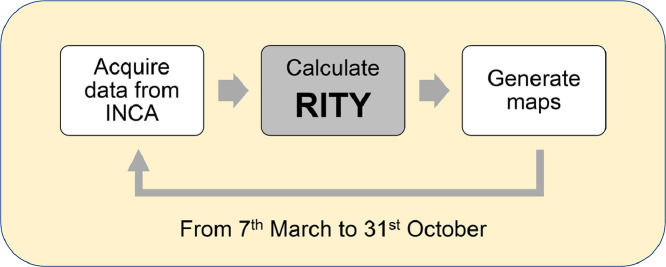

**Table:** Specification table

Subject area:	Agricultural and Biological Sciences
More specific subject area:	Forest ecology and management
Method name:	RITY
Name and reference of original method:	Original method name: PHENIPS Reference of original method: Baier, P., Pennerstorfer, J., Schopf, A., 2007. PHENIPS—A comprehensive phenology model of *Ips typographus* (L.) (Col., Scolytinae) as a tool for hazard rating of bark beetle infestation. Forest Ecol. Manag. 249, 171–186. https://doi.org/10.1016/j.foreco.2007.05.020 New method name: RITY Reference of new method: Ogris, N., Ferlan, M., Hauptman, T., Pavlin, R., Kavčič, A., Jurc, M., De Groot, M., 2019. RITY – A phenology model of *Ips typographus* as a tool for optimization of its monitoring. Ecol. Model. 410, 108,775. https://doi.org/10.1016/j.ecolmodel.2019.108775
Resource availability:	There are no special resources. The original methods mentioned above can be used to reproduce the method.

## Method details

European spruce bark beetle, *Ips typographus* (L.), is one of the most economically important forest pests in Europe [1]. Management of European spruce bark beetle requires continuous population monitoring. Additionally, to assess the likelihood of mass outbreaks in due time, appropriate monitoring tools need to accurately predict the actual developmental process of the bark beetle population and the number of bark beetle generations per year. Such a tool or model would need to address the spatiotemporal dynamics of the bark beetle population and its temperature-dependant phenology, including timing and the number of all filial and sister generations. One model that meets all of these criteria is the PHENIPS model developed by Baier et al. [2]. The model was validated for a Central European population of *I. typographus* and has been used as an online tool for predicting bark beetle development in Austria [3] and Germany [4]. PHENIPS has also been independently validated in the Czech Republic [5] and included in various risk assessment frameworks [6], [7], [8], [9], [10].

PHENIPS was implemented, calibrated, validated and improved for use in Slovenian forestry. Through this process, a new model was developed with a different name: RITY-2 [11]. There are several differences between RITY-2 and PHENIPS. The main difference is in the estimation of air and bark temperature. PHENIPS uses a topoclimatic model with the estimated solar radiation and elevation of the location as input variables to calculate daily mean and maximum air temperature [2]. RITY-2 uses daily minimum, mean and maximum air temperatures from the INCA system (Integrated Nowcasting through Comprehensive Analysis) [12,13] and corrects these temperatures with linear models to simulate the air temperature in the forest (Eqs. (1)–(3)) [11]. Calculation of bark temperature in PHENIPS is based on regression analysis, with air temperature and solar radiation as predictive variables [2], whereas RITY-2 uses linear models with air temperature as the sole input variable (Eqs. (4)–(6)) [11]. The models also differ with respect to the method for calculating the daily effective bark temperature. RITY-2 uses linear and nonlinear functions (Eqs. (7)–(9)) to calculate daily effective bark temperature [11], whereas PHENIPS uses the estimated difference between the linear and nonlinear functions for temperatures above the optimum temperature [2]. The models are similar with respect to the calculation of the phenology of *I. typographus*. However, the models use different thresholds for the onset of swarming and infestation. RITY-2 uses 53.0 dd and 155.6 dd, respectively (Eqs. (10) and (11)) [11], while PHENIPS uses 60.5 dd and 140.3 dd, respectively [2]. PHENIPS records the thermal sum from 1st April onwards. RITY-2 starts the calculation on 7th March. The models also differ with respect to the daily maximum air temperature threshold (ATmax) for the onset of different phenological events (swarming, infestation, filial broods, sister broods). In PHENIPS this threshold is 16.5 °C, while in RITY-2 it is 14.5 °C. Additionally, RITY-2 distinguishes between the daily maximum air temperature (ATmax) in the forest and the estimated daily maximum air temperature given by the INCA system (I_max_).

RITY-2 includes a prediction of the onset of spring swarming, onset of infestation, re-emergence of parental beetles, and the number and emergence time of filial and sister broods. We introduced an innovative procedure that finds the most appropriate spring date threshold from which the calculation of the phenological model is initiated and that improves the accuracy of the phenological model in estimating the onset of swarming and onset of infestation. Furthermore, INCA enables simulations and predictions of *I. typographus* phenology with high enough spatial and temporal resolution to take into account local differences in climate variability. The phenology of *I. typographus* is simulated with three scenarios using minimum, mean and maximum daily temperatures, labelled as the MIN, AVG and MAX scenarios, respectively. Therefore, RITY-2 and PHENIPS are comprehensive and much more complex models in comparison to earlier models where only the linear relation between developmental rate and ambient temperature above the lower developmental threshold was used to estimate the cumulative sum of effective temperatures required to complete *I. typographus* development [14], [15], [16], [17].

**Fig. 1:**
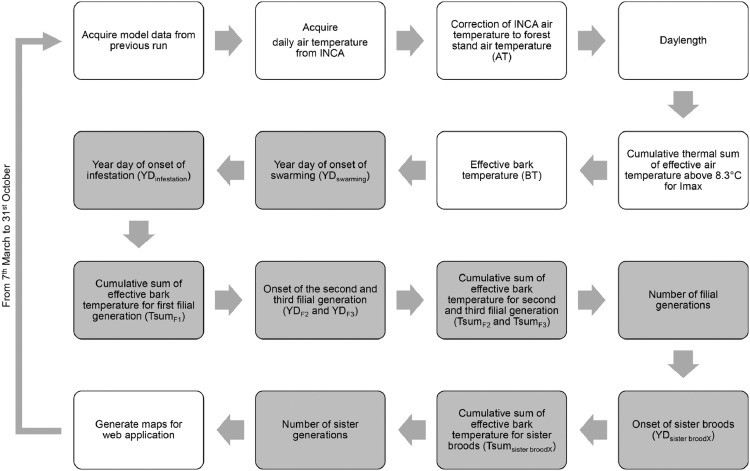
Diagram of the RITY-2 model. The model is calculated stepwise and every day from 7th March to 31st October. White boxes are preliminary steps and grey boxes depict core computation steps [11].

The purpose of this paper is to describe the calculation procedure for RITY-2. The model is calculated stepwise and every day from 7th March to 31st October. A conceptual diagram summarizing the main steps of the RITY-2 model calculation is presented in Fig. 1. A detailed description of the model computation along with formulae is given in the following text.

## Procedure for finding the most appropriate spring date threshold from which the calculation of the phenological model is initiated

PHENIPS set 1st April as the date threshold for the calculation of this thermal sum [2]. However, this might not be the most appropriate date for other regions. Therefore, before the application of PHENIPS to other regions, the spring date threshold from which the calculation of the phenological model is initiated must be calibrated and validated. The latest date for calculation of the cumulative thermal sum for the onset of spring swarming and host tree colonization is determined with an iteration procedure in which the lowest mean absolute error of the predicted onset of spring swarming and host tree colonization is achieved [11]. In the case of Slovenia, the spring date threshold was determined to be 7th March [11].

## Air temperature, bark temperature and effective bark temperature

Minimum, mean and maximum daily air temperatures (AT) in the forest stand are calculated using linear models and daily air temperature from the INCA system (I_min_, I_mean_, I_max_) for each location *x* and day *i* (*x_i_*):

(1) $$\text{ATmin}\left(x_{i}\right)=1.44+0.82\times I_{\text{min}}\left(x_{i}\right)$$

(2) $$\text{ATmean}\left(x_{i}\right)=0.50+0.81\times I_{\text{mean}}\left(x_{i}\right)$$

(3) $$\text{ATmax}\left(x_{i}\right)=1.03+0.86\times I_{\text{max}}\left(x_{i}\right)$$

Minimum, mean and maximum bark temperatures (BT) are calculated using linear models and daily estimated air temperatures:

(4) $$\text{BTmin}\left(x_{i}\right)=0.56+0.99\times \text{ATmin}\left(x_{i}\right)$$

(5) $$\text{BTmean}\left(x_{i}\right)=-0.48+1.03\times \text{ATmean}\left(x_{i}\right)$$

(6) $$\text{BTmax}\left(x_{i}\right)=0.03+0.99\times \text{ATmax}\left(x_{i}\right)$$

Effective bark temperature (BTeff) between the lower development threshold (DT_L_ = 8.3 °C) and the optimum temperature (*T*_O_ = 30.4 °C) is calculated with a linear function (7). Effective bark temperature between *T*_O_ and the upper development threshold (DT_U_ = 38.9 °C) is calculated with a non-linear function (8). For temperatures outside the lower and upper temperature thresholds (*T* ≤ DT_L_ and *T* ≥ DT_U_), BTeff is set to zero (9). Three variants of BTeff are calculated with Eqs. (4)–(6).

(7) $$\text{If}\text{BT}\left(x_{i}\right)>DT_{L}\text{and}\text{BT}\left(x_{i}\right)\leq T_{O}\;\text{BTeff}\left(x_{i}\right)=\text{BT}\left(x_{i}\right)-8.3$$

(8) $$\begin{array}{ccc} & & \text{If}\text{BT}\left(x_{i}\right)>T_{O}\text{and}\text{BT}\left(x_{i}\right)<DT_{U}\;\text{BTeff}\left(x_{i}\right)=\left(T_{O}-DT_{L}\right)\times \left(\text{exp}(\alpha \times \text{BT}\right)-\text{exp}\left(\alpha \times T_{\max}-\left(T_{\max}-\text{BT}\right)/\beta \right)-\gamma ),\\ & & \text{where}\alpha =0.02876507;\beta =3.5922336;\gamma =1.24657367;T_{\text{max}}=40.9958913 \end{array}$$

(9) $$\text{If}\text{BT}\leq DT_{L}\text{or}\text{BT}\left(x_{i}\right)\geq DT_{U}\;\text{BTeff}\left(x_{i}\right)=0$$

## Phenology model of *I. typographus*

RITY-2 comprises the following stepwise computations for any location (grid cell *x*) that are computed every day from 7th March to 31st October:

• Calculation of the year day of the onset of swarming (YD_swarming_) based on the threshold for flight activity (14.5 °C) and on cumulative daily maximum air temperatures (I_max_) above DT_L_ from 7th March onwards:
  (10) $$YD_{\text{swarming}}\;\text{if}\;I_{\text{max}}>14.5^{\circ }C\;\text{and}\;\sum \left(I_{\text{max}}-8.3\right)\geq 53.0\;\text{dd}$$
• Calculation of the year day of the onset of infestation (YD_infestation_) based on the threshold for flight activity and on cumulative daily maximum air temperatures (I_max_) above DT_L_ from 7th March onwards:
  (11) $$YD_{\text{infestation}}\;\text{if}\;I_{\text{max}}>14.5^{\circ }C\;\text{and}\;\sum \left(I_{\text{max}}-8.3\right)\geq 155.6\;\text{dd}$$
• Calculation of the cumulative sum of effective bark temperature (Tsum) relative to the thermal sum required for total development (*K* = 557 dd) of the first filial generation (F1):
  (12) $$\text{if}\text{YD}\geq YD_{\text{infestation}}\;\text{Tsu}m_{F1}=K^{-1}\times \sum \text{BTeff}$$
• Calculation of the onset of sister broods (YD_S_*_j_*) and their cumulative sum of effective bark temperature (BTeff) relative to the thermal sum (Tsum) required for total development (*K* = 557 dd) of the *j*th sister brood (S*j*):
  (13) $$\begin{array}{c} YD_{Sj}\text{if}\text{Tsu}m_{F1}>j-0.5\text{and}I_{\text{max}}>14.5^{\circ }C\text{and}\text{daylength}\geq 14.5h\\ \text{if}\text{YD}\geq YD_{Sj}\;\text{Tsu}m_{\mathrm{Sj}}=K^{-1}\times \sum \text{BTeff} \end{array}$$
• Calculation of the onset of the second (YD_F2_) and third filial (YD_F3_) generation and their cumulative sum of effective bark temperature (Tsum_Fx_) relative to the thermal sum required for total development (*K* = 557 dd):
  (14) $$\begin{array}{c} YD_{F2}\text{if}\text{Tsu}m_{F1}>1\text{and}I_{\text{max}}>14.5^{\circ }C\text{and}\text{day}\text{length}\geq 14.5h\\ \text{if}\text{YD}\geq YD_{F2}\;\text{Tsu}m_{F2}=K^{-1}\times \sum \text{BTeff} \end{array}$$
  (15) $$\begin{array}{c} YD_{F3}\text{if}\text{Tsu}m_{F1}>2\text{and}I_{\text{max}}>14.5^{\circ }C\text{and}\text{day}\text{length}\geq 14.5h\\ \text{if}\text{YD}\geq YD_{F3}\;\text{Tsu}m_{F3}=K^{-1}\times \sum \text{BTeff} \end{array}$$

Finally, the relative thermal sum of each initiated generation at the end of October of each year and of each grid cell is evaluated for its probability of survival during the following cold period. At a relative thermal sum higher than 60% of the thermal sum required for total development (Tsum_F_*_x_* ≥ 0.6), the initiated generation has completed its preimaginal development and can successfully hibernate as young adults. Therefore, initiated generations with relative thermal sums < 0.6 are ignored when calculating the total number of potential generations per year. Additionally, the model is calculated only for grid cells containing appropriate host trees (Norway spruce) for *I. typographus*.

**Fig. 2:**
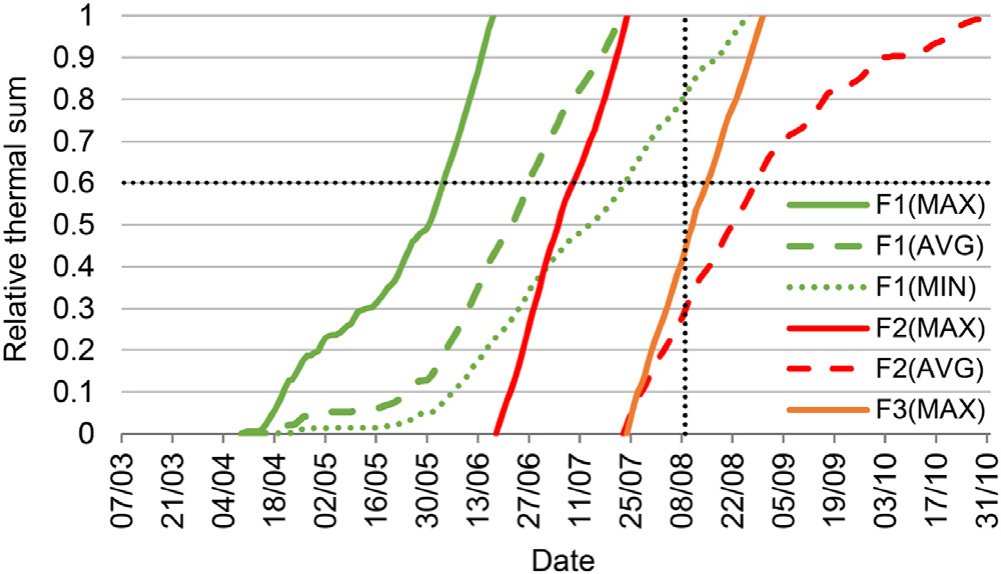
Example of a chart produced by the point-based web application [18]. Estimated relative thermal sums of the predicted development of *I. typographus* using INCA temperature data at Bled (coordinates: 14.11419E, 46.36871N) in 2019 according to the MIN, AVG and MAX scenarios for three filial generations (Fx). Development of a new generation starts at the predicted onset of infestation and later at the emergence of filial beetles of the previous generation. The dotted horizontal line marks the threshold for successful hibernation (relative thermal sum ≥ 0.6); the dotted vertical line indicates the threshold for induction of diapause (day length = 14.5 h). Developmental stages can be determined by the following range of relative thermal sums: egg [0, 0.1], larvae [0.1, 0.5], pupae [0.5, 0.6], teneral adult [0.6, 1.0].

**Fig. 3:**
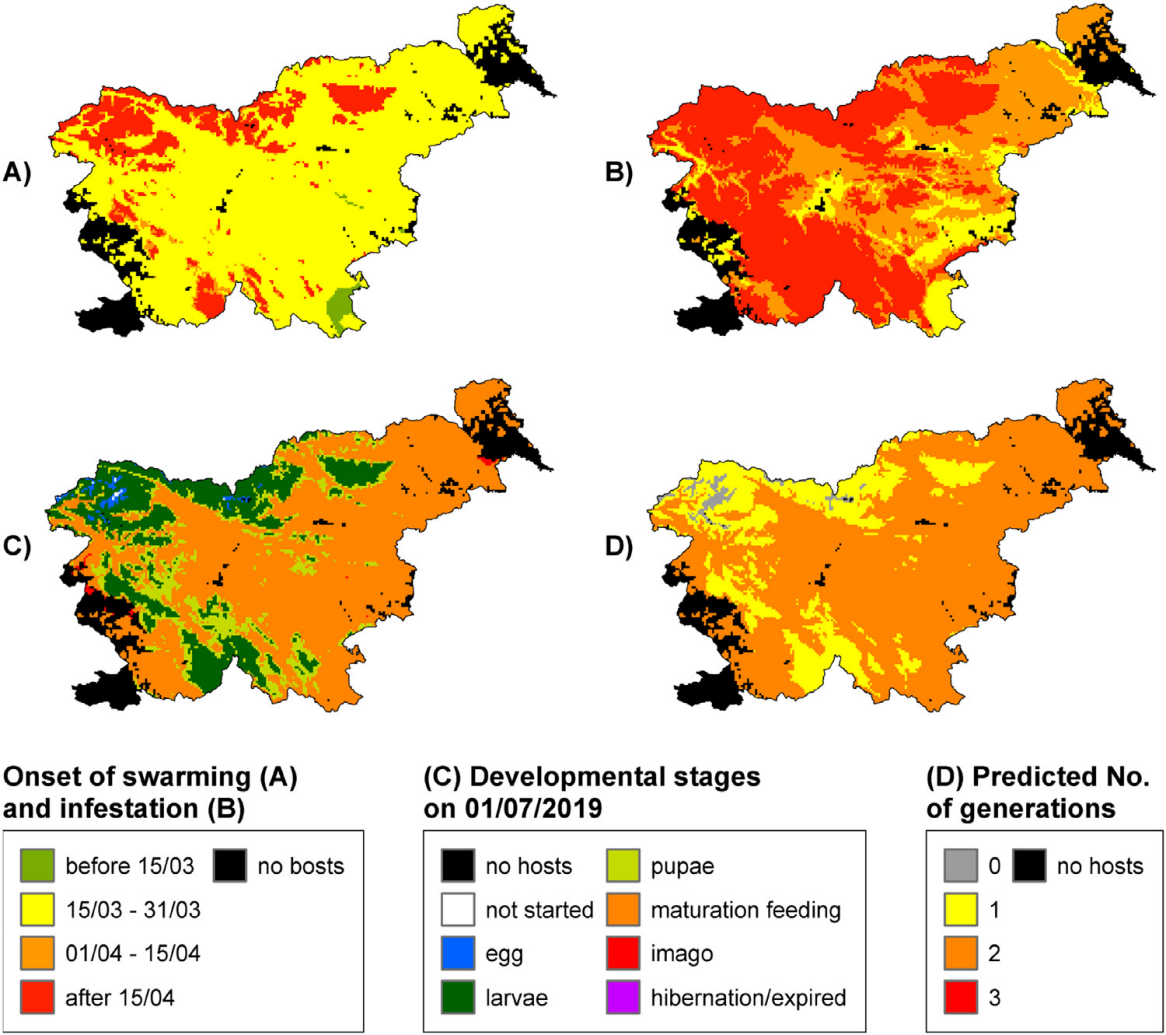
Examples of maps produced by the spatial-based web application [19]. Onset of swarming (A), onset of infestation (B), developmental stages on 01/07 (C), and predicted number of generations (D) for 2019 in Slovenia according to the AVG scenario.

We developed two public web applications for RITY-2. The first is point-based and produces a chart of the relative thermal sums of the predicted development of *I. typographus* according to the MIN, AVG and MAX scenarios for any location in Slovenia [18] (Fig. 2). The second web application is spatial and produces several maps in a time series of the predicted development of *I. typographus* for the whole region of Slovenia [19]. The maps show the onset of spring swarming, onset of infestation of different broods, development of developmental stages of spruce bark beetle for several filial and sister broods, and number of filial and sister broods for any chosen date (Fig. 3). The source codes for both applications are publicly available [20,21].
